# Early changes in right ventricular longitudinal function in chronic asymptomatic alcoholics revealed by two-dimensional speckle tracking echocardiography

**DOI:** 10.1186/s12947-016-0058-3

**Published:** 2016-04-19

**Authors:** Sisi Meng, Lijuan Guo, Guangsen Li

**Affiliations:** 1 Department of Ultrasound, Second Affiliated Hospital of Dalian Medical University, Dalian, 116027 China; 2 Department of Echocardiography, Liaoning Provincial People’s Hospital, Shenyang, 110000 China

**Keywords:** Two-dimensional speckle tracking echocardiography, Alcoholics, TAPSE, Right ventricular function

## Abstract

**Background:**

Heart ventricular dysfunction has been characterized as reduced longitudinal function of the right ventricle (RV), and is associated with chronic alcohol abuse. This study investigated the use of two-dimensional speckle tracking echocardiography (2DSTE) to assess the longitudinal systolic and diastolic RV function of patients with alcoholic myocardial damage.

**Methods:**

We stratified 92 asymptomatic alcoholic men into three groups of increasing alcohol intake, Groups A–C. Thirty age-matched normal adult men served as the control group. Conventional echocardiography and tricuspid annulus peak systolic excursion (TAPSE) parameters were obtained. 2DSTE parameters were recorded from an apical 4-chamber view of the RV free wall. LV peak global longitudinal systolic strain was calculated from segmental averaging of the three apical long-axis views.

**Results:**

In Group C, the RV end diastolic diameter (RVEDD) was dramatically higher than that of Groups A, B and the control, while TAPSE was significantly lower in Group C compared with the other experimental groups. In Group B, the longitudinal early diastolic strain rate (SRe) and late diastolic strain rate (SRa) of the RV free wall, and LV longitudinal strain were significantly lower than that of Group A or the control. In Group C, all the 2DSTE parameters were significantly lower than that of the other groups. A significant negative linear correlation was noted between global RV systolic parameters systolic strain peak (S), peak systolic strain rate (SRs) and TAPSE (*r*_1_=−0.84, *r*_2_=−0.72, respectively, *P* <0.05).

**Conclusions:**

Two-dimensional STE provided an effective and non-invasive method to assess the RV longitudinal function of patients with alcoholic myocardial damage. This methodology may be useful for diagnosing, directing treatment, and judging prognosis of alcoholic cardiac damage.

## Background

Alcohol is a risk factor of heart disease, and long-term alcohol abuse may have detrimental effects on ventricular function [1, 2], possibly leading to alcoholic cardiomyopathy (ACM). Recent studies have indicated that changes in heart dimensions and function are common in alcoholics [3].

Longitudinal LV dysfunction is one of the earliest biomarkers of ventricular damage [4, 5]. Two-dimensional speckle tracking echocardiography (2DSTE) reportedly can sensitively assess left ventricular (LV) function and predict early pathogenesis associated with myocardial dysfunction [6, 7].

Although the prognostic value of right ventricular (RV) systolic and diastolic function has been documented for cardiac symptoms and exercise capacity [8], prior studies using 2DSTE have focused on LV structure and function [9, 10], and studies on RV function are few. RV function is difficult to assess quantitatively using traditional imaging because of the thin RV myocardial wall and complex structure of the RV myocardial wall, thus it is usually described only qualitatively [11, 12].

The limitations of other echocardiographic techniques are overcome with 2DSTE, as the focus is on endocardial structures interference from the RV wall is avoided. Prior studies have suggested that RV dysfunction can be determined quantitatively by the change in longitudinal movement of the RV, relative to that of the normal RV [13, 14]. Since the subendocardial layer of the myocardium is the most sensitive to alcohol damage [15], we hypothesized that measurements of RV longitudinal deformation could be a sensitive measure of RV function in an alcoholic population [16].

In this setting, we hypothesized that 2DSTE could be used for early evaluation of changes in RV longitudinal function in patients with alcoholic myocardial damage. While there have been several studies of deformation that have touched upon this topic, none have used 2D-STE to study the RV.

## Methods

### Patient population

Ninety-two male asymptomatic alcoholics, 42–65 years old, were examined between June 2009 and March 2013 and served as the experimental group. These patients were categorized into three groups according to the amount and duration of alcohol consumption: Group A, ≥90 mg ethanol or ≥2–3 bottles of beer, 3–5 days/week for 5–8 years; Group B, ≥90 mg ethanol or ≥2–3 bottles of beer, 3–5 days/week for 9–20 years; and Group C, ≥150 mg ethanol or ≥4 bottles of beer, 6–7 days/week for >10 years, which met the standard for diagnosis of alcoholic cardiomyopathy (ACM) [17].

Thirty age-matched (40–65 years) healthy male volunteers served as the control group (Group D). Control subjects did not exhibit any cardiovascular abnormalities.

The exclusion criteria included a history of congenital heart disease, hypertension, diabetes, ischemic or vascular heart diseases, rhythm problems and atrial fibrillation, pulmonary hypertension, systemic and metabolic diseases that could adversely affect cardiac structure and function such as valvular heart disease, or using cardiac medications.

All procedures were carried out in accordance with institutional guidelines. The local medical ethics committee (Second Affiliated Hospital of Dalian Medical University, Dalian, China) approved the study, and all patients provided written informed consent before entering the study.

### Instruments and methods

Recordings were obtained with a GE Vingmed Vivid 7 (GE Vingmed Ultrasound, Horten, Norway) scanner equipped with a 1.7–3.4 MHz transducer (M3S probe). Standard echocardiography and 2DSTE measurements of the RV and LV were analyzed off-line using dedicated software (EchoPAC, GE Vingmed Ultrasound).

### Two-dimensional and M-mode echocardiographic acquisition

The examination focused on the measurement of LV and RV dimensions. The end diastolic diameters, ejection fractions, and tricuspid annulus peak systolic excursion (TAPSE) were captured as follows. RV and LV end diastolic diameters (RVEDD and LVEDD, respectively) were measured in the parasternal long-axis view. From the apical four-chamber view, the LV ejection fraction (LVEF) was obtained using a commercially available software program that applied the biplane Simpson’s method in the four-chamber view. The RV ejection fraction (RVEF) was calculated from the RV fractional area change.

TAPSE acquisition was performed by M-mode echocardiography in the four-chamber view: an M-mode cursor was steered through the lateral portion of the tricuspid annulus, which delineates the RV atrioventricular plane. The magnitude of the tricuspid valve ring along the M-mode line was determined by measuring the maximal distance between the leading edge of the highest and lowest echoes during each cardiac cycle.

### 2DSTE acquisition

Two-dimensional dynamic images were recorded for subsequent analyses. High frame rate acquisition was used (≥100 frames/s). At least three cardiac cycles were stored for offline analysis. All 2DSTE data were measured by averaging data over three heartbeats.

A crucial part of the analysis was based on the correct adjudication of event timing. For example, end-diastole was defined at the peak R of the electrocardiographic QRS complex, and end-systole was defined as the first negative crossover of the velocity curve. Offline analysis was performed using a special software program (EchoPAC 6.1.0 GE Vingmed).

Out of the cardiac cycles recorded, the RV endocardial surface was manually defined using the clearest (i.e., least myocardial artifact) of apical 4-chamber views. To train the software to track automatically the relevant myocardial motion, a region of interest was manually defined on the RV wall. Myocardial deformation was defined as longitudinal strain and strain rate was examined at basal, middle, and apical segments of the RV wall, including peak systolic displacement (P), systolic strain peak (S), peak systolic strain rate (SRs), peak early diastolic strain rate (SRe) and late diastolic strain rate peak (SRa).

In addition, LV peak global longitudinal systolic strain was calculated from segmental averaging of the three apical long-axis views (4-chamber, 2-chamber, and apical long-axis). The LV region of interest was defined by tracking the endocardial and epicardial borders, and adjusted to accommodate wall thickness.

### Statistical analysis

All analyses were performed using SPSS version 17.0 for Windows (SPSS, Chicago, IL, USA). Variables are presented as mean ± standard deviation. One-way analysis of variance was used to assess changes among the four groups. Multiple comparisons between the groups were performed using the Student–Newman–Keuls method. Correlation coefficients, 95 % confidence intervals (CIs) and percentage errors were reported. A *P*-value <0.05 was considered statistically significant. Intra-observer analysis of RV longitudinal strain in the 4-chamber view was conducted 2 months after completion of the initial measurements (by author MSS). For inter-observer variability, a second observer (LGS) analyzed 20 % of the initial images. Intra-observer variability and inter-observer variability were assessed using the intra-class correlation coefficient (ICC) [18].

## Results

### General characteristics and LV echocardiographic parameters

All study subjects were in sinus rhythm at rest, and none had a history of cardiac surgery. The four groups showed an average distribution in age, body weight, height, body mass index and body surface area, which were similar among the groups (*P* >0.05; Table 1). Conventional echocardiographic findings revealed that the LV dimensions, LV mass index, and greater ventricle wall thickness was significantly greater in Group C compared with that of the other groups (*P* <0.05).

**Table 1:** Demographics and LV echocardiographic parameters

		Control (*n* = 30)	A (*n* = 30)	B (*n* = 31)	C (*n* = 31)
Demographics	Age, y	50.3 ± 8.4	51.5 ± 6.0	49.4 ± 3.7	52.3 ± 7.6
	BSA, m^2^	1.56 ± 0.26	1.62 ± 0.13	1.53 ± 0.34	1.66 ± 0.23
	BMI, kg/m^2^	26.2 ± 2.8	25 .4 ± 3.3	26.4 ± 1.9	26.1 ± 2.1
	SBP, mmHg	129.4 ± 6.2	120.5 ± 11.7	125.3 ± 5.8	131.5 ± 6.7
	DBP, mmHg	77.3 ± 5.2	79.5 ± 5.2	78.2 ± 5.0	80 .3 ± 5.2
	HR, bpm	68.5 ± 3.4	70.5 ± 5.6	62.5 ± 5.3	67.5 ± 2.6
LV parameters	LVEF, %	61.20 ± 2.86	60.65 ± 3.03	58.25 ± 5.64	42.15 ± 6.77^a,b,c^
	FS, %	34.6 ± 1.02	33.4 ± 2.71	30.2 ± 3.43	21.6 ± 1.25^a,b,c^
	LVDD, mm	47.46 ± 2.22	48.94 ± 3.40	49.40 ± 4.45	60.04 ± 3.61^a,b,c^
	LVDs, mm	31.01 ± 4.47	32.70 ± 4.06	33.37 ± 4.19	44.28 ± 4.83^a,b,c^
	IVSd, mm	9.47 ± 0.62	9.53 ± 0.34	9.84 ± 0.86	12.01 ± 0.45^a,b,c^
	PWd, mm	9.30 ± 0.61	9.37 ± 0.50	9.79 ± 0.88	11.76 ± 0.30^a,b,c^

*BMI,* body mass index, *BSA* body surface area, *DBP* diastolic blood pressure, *SBP* systolic blood pressure, *LVEF* left ventricular ejection fraction, *FS* Left ventricular fractional shortening, *LVDD* left ventricular end-diastolic dimension, *LVDs* left ventricular end-systolic dimension, *IVSD* Interventricular septum diastolic thickness diameter, *PWD* posterior wall diastolic thickness diameter, ^a^ *P* <0.01 cf. control, ^b^ *P* <0.01 cf. Group A; ^c^ *P* <0.01 cf. Group B

### RV echocardiographic parameters

The RVEDD of Group C was significantly higher than that of the other groups, and the TAPSE was significantly lower (*P* <0.05, both; Table 2). Furthermore, the RVEF of Group C was lower than that of the control group, although this did not reach statistical significance (*P* >0.05).

**Table 2:** RV echocardiographic parameters

	Control (*n* = 30)	A (*n* = 30)	B (*n* = 31)	C (*n* = 31)
RVEF, %	59.75 ± 5.62	59.44 ± 3.71	55.13 ± 3.90	42.14 ± 4.29
RVEDD, mm	23.87 ± 1.82	24.15 ± 1.56	24.23 ± 1.49	26.20 ± 2.13^a,b,c^
TAPSE, mm	21.5 ± 2.17	19.9 ± 2.01	16.4 ± 2.22	12.2 ± 1.98^a,b,c^

*RVEF* right ventricular ejection fraction, *RVEDD* right ventricular end-diastolic dimension, *TAPSE* tricuspid annulus peak systolic excursion. ^a^ *P* < 0.01 cf. control; ^b^ *P* < 0.01 cf. Group A; ^c^ *P* < 0.01 cf. Group B

### 2DSTE parameters

**Fig. 1:**
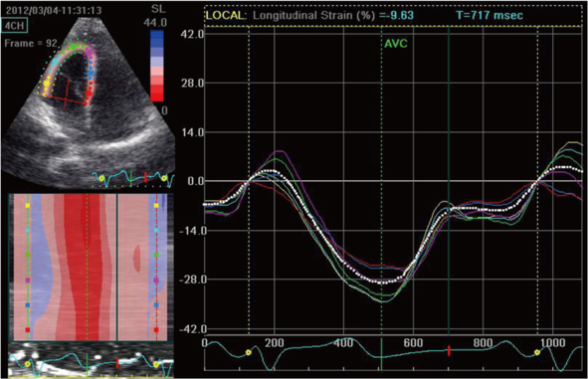
Right ventricular peak systolic longitudinal strain of the control group

**Fig. 2:**
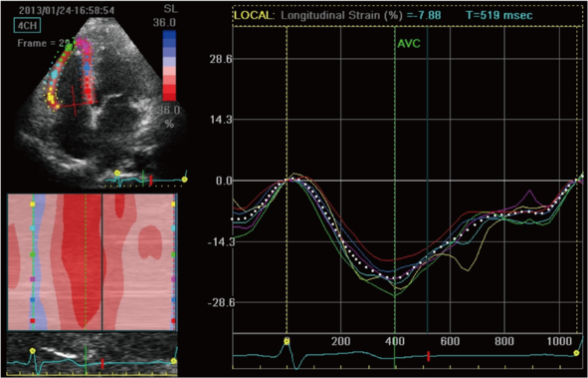
Right ventricular peak systolic longitudinal strain of Group A

**Fig. 3:**
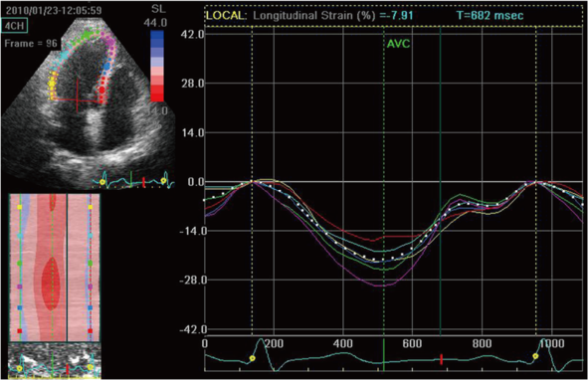
Right ventricular peak systolic longitudinal strain of Group B

**Fig. 4:**
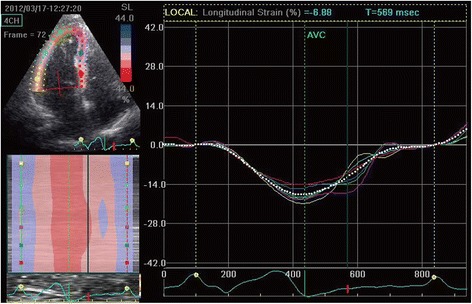
Right ventricular peak systolic longitudinal strain of Group C

The LV longitudinal strain was less in Group B and C compared with both Group A and the control (Table 3). The average longitudinal strain and strain rate of each segment in the basal, mid, and apical regions of the RV free wall were determined in each group (Table 4). All measured echocardiographic variables in Group A were similar to that of the control group (*P* >0.05). The SRe and SRa of each RV free wall segment were significantly lower in Group B than in either Group A or the control group (*P* <0.05). However, the parameters P, S, and SRs of Groups A, B, and the control group were similar (*P* >0.05). Notably, all the diastolic parameters and the systolic parameters P, S, and SRs were significantly lower in Group C than in the other groups (*P* <0.05, all). Changes in myocardial strain within each segment (basal, mid, and apical) between the four groups are presented in Figs. 1, 2, 3 and 4.

**Table 3:** 2DSTE parameters of LV peak global longitudinal systolic strain

	Control (*n* = 30)	A (*n* = 30)	B (*n* = 31)	C (*n* = 31)
Basal	−19.4 ± 4.1	−19.2 ± 5.2	−15.3 ± 5.06^ab^	−9.5 ± 4.7^abc^
Middle	−20.9 ± 4.2	−21.7 ± 4.5	−16.3 ± 5.2^ab^	−11.4 ± 6.3^abc^
Apical	−24.6 ± 5.1	−24.2 ± 4.2	−19.4 ± 4.7^ab^	−13.9 ± 7.3^abc^

^a^ *P* <0.05 cf. control; ^b^ *P* <0.05 cf. Group A; ^c^ *P* <0.05 cf. Group B

**Table 4:** 2DSTE parameters of RV free wall

		Control (*n* = 30)	A (*n* = 30)	B (*n* = 31)	C (*n* = 31)
P, mm	Base	21.34 ± 1.89	19.91 ± 0.78	15.14 ± 3.32	8.78 ± 2.22 ^a,b,c^
	Mid	13.61 ± 1.14	12.58 ± 0.99	10.29 ± 2.70	5.99 ± 1.73 ^a,b,c^
	Apex	5.09 ± 0.97	4.55 ± 0.72	3.36 ± 0.63	2.08 ± 0.92 ^a,b,c^
S, %	Base	32.19 ± 7.38	26.85 ± 2.80	18.12 ± 3.33	10.71 ± 2.71 ^a,b,c^
	Mid	29.09 ± 7.30	24.46 ± 3.09	19.19 ± 2.63	12.29 ± 4.62 ^a,b,c^
	Apex	27.51 ± 2.47	25.45 ± 2.21	21.20 ± 1.86	17.53 ± 5.02 ^a,b,c^
SRs, s^−1^	Base	1.62 ± 0.28	1.29 ± 0.10	1.15 ± 0.10	0.76 ± 0.13 ^a,b,c^
	Mid	1.45 ± 0.27	1.23 ± 0.08	1.12 ± 0.11	0.87 ± 0.13 ^a,b,c^
	Apex	1.57 ± 0.25	1.28 ± 0.18	1.09 ± 0.11	0.89 ± 0.11 ^a,b,c^
SRe, s^−1^	Base	2.06 ± 0.15	2.00 ± 0.14	1.69 ± 0.08 ^a,b^	1.35 ± 0.11 ^a,b,c^
	Mid	1.98 ± 0.09	1.95 ± 0.13	1.71 ± 0.07 ^a,b^	1.32 ± 0.13 ^a,b,c^
	Apex	2.11 ± 0.14	2.02 ± 0.13	1.82 ± 0.07 ^a,b^	1.31 ± 0.25 ^a,b,c^
SRa, s^−1^	Base	1.58 ± 0.16	1.39 ± 0.13	1.06 ± 0.71 ^a,b^	0.88 ± 0.08 ^a,b,c^
	Mid	1.57 ± 1.40	1.50 ± 0.12	1.39 ± 0.22 ^a,b^	0.99 ± 0.24 ^a,b,c^
	Apex	1.53 ± 0.16	1.40 ± 0.21	1.17 ± 0.30 ^a,b^	0.66 ± 0.15 ^a,b,c^

*P* peak systolic displacement, *S* systolic strain peak, *SRs* peak systolic strain rate, *SRe* peak early diastolic strain rate, *SRa* late diastolic strain rate peak. ^a^ *P* <0.01 cf. control, ^b^ *P* <0.01 cf. Group A, ^c^ *P* <0.05 cf. Group B

### Tapse

**Fig. 5:**
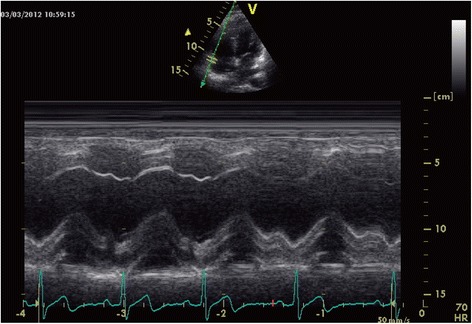
TAPSE of control group

**Fig. 6:**
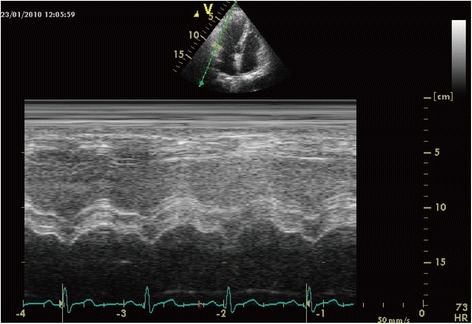
TAPSE of Group C

**Fig. 7:**
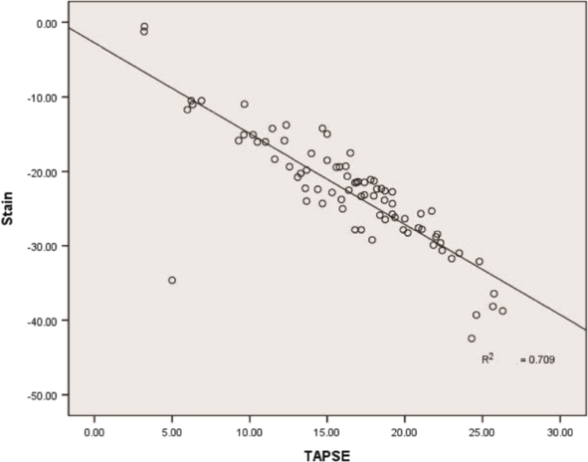
Correlation between TAPSE and right ventricular peak systolic longitudinal strain

TAPSE was significantly lower in Group C compared with that of the other experimental groups (Figs. 5 and 6). A significant negative linear correlation was also noted between global RV systolic parameters S and SRs and TAPSE (*r*_1_=−0.84, *r*_2_=−0.72, respectively, *P* <0.05; Fig. 7).

Using 2D-STE to evaluate alcoholic cardiac damage, inter- and intra-observer results had good reproducibility and small variability (Table 5).

**Table 5:** Inter and intra-observer analyses for RV strain

	Intra-observer				Inter-observer			
	R	bias (%)	LOA (%)	ICC	R	bias (%)	LOA (%)	ICC
P	0.86	0.93	−1.26 ~ 2.24	0.960	0.84	0.81	−1.79 ~ 2.89	0.949
S	0.76	−3.37	−9.56 ~ 6.24	0.824	0.84	−3.19	−10.79 ~ 4.89	0.830
SRs	0.89	0.65	−1.13 ~ 2.15	0.973	0.90	0.59	−1.09 ~ 2.05	0.986
SRe	0.80	2.23	−6.14 ~ 5.23	0.866	0.78	2.72	−8.76 ~ 2.21	0.825
SRa	0.74	3.67	−11.88 ~ 8.70	0.810	0.76	3.98	−12.69 ~ 9.22	0.805

*R*, coefficient of determination, *LOA* limit of agreement, *ICC* intra-class correlation coefficient

## Discussion

Ventricular dysfunction is consistent with the sympathetic depressant effect of high alcohol consumption. Findings of reduced longitudinal function in the RV have been shown to reflect global ventricular dysfunction [14, 19]; further, RV dilatation and dysfunction are common findings in the pathogenesis of ACM and are closely related to the severity of LV disease [20, 21]. In addition, some studies have suggested that fat replacement of the RV in patients with ACM was likely associated with ACM [22], and that RV dysfunction was an independent predictor of cardiac death. The effect of cardiac growth on strain, changing loading conditions and ventricular contractility, myocardial fiber disarray, fat accumulation cardiac interstitial fibrosis, and edema seen in alcoholics can all affect myocardial strain.

Anatomically, the structure of the RV is more complex than that of the LV, appearing triangular when viewed from the side and crescentic when viewed in cross-section. RV myocardial fibers are three times thinner than LV fibers, making the RV myocardial wall relatively thin. The RV wall is mainly composed of two layers: the superficial layer fibers that are aligned circumferentially and parallel to the AV groove, and the deep layer fibers arranged longitudinally, from base to apex, which predominate RV contractile motion. Importantly, RV and LV share a common interventricular septum and the epicardial fibers are mutually encircled, thus ventricular interdependence cannot be overlooked.

Due to the complex shape of the RV, conventional transthoracic echocardiography assessment of the RV is nearly always based on qualitative estimation [23]. Thus, further studies are warranted to delineate RV function using advanced imaging techniques. Recently, a new technique has been developed for 2D-STE using B-mode images. Using this technique, myocardial deformation can be assessed by acoustic irregularities in the speckled myocardial patterns. In general, these patterns are considered stable and changes in speckle position indicate myocardial motion. Changes between the speckles are interpreted as myocardial deformation. Validation studies on 2D-STE suggest that the method is reliable and angle-independent and is not affected by translation or tethering from the surrounding tissue [24, 25].

To assess ventricular function in a variety of different cardiovascular diseases, 2D-STE has been confirmed to be a feasible and sensitive method [26–29]. Estimation of cardiac function by 2DSTE, without an LV geometric model, may detect abnormal RV motion prior to the occurrence of adverse systemic effects [30]. Moreover, 2D-STE is a more reliable and comprehensive assessment of myocardial function compared with tissue Doppler imaging (TDI) [31], which depends on the direction of the Doppler angle for accurate measurement.

Because circulatory support functions of the RV are performed by longitudinal muscle, some researchers have suggested that peak longitudinal strain could be a biomarker for assessment of RV function [32]. Further, longitudinal measurements have the highest reproducibility and smallest bias, compared with the radial or circumferential dimensions [33], making longitudinal measurements a suitable approach to assess RV function. Salerno et al. [34] report the use of 2DSTE to evaluate the RV function in early dilated cardiomyopathy (DCM). Although only the RVEDD was slightly higher, 2DSTE could detect the regional RV myocardial damage. Our data is in agreement with results from this previous study on RV dysfunction.

Compared with the control group, the RVEDD of patients in Group C was significantly higher, based on conventional ultrasound parameters. However, 2D-STE analysis of Re and SRa in Group B were significantly lower than in Group A and the control, indicating altered diastolic RV function. This finding suggests that 2DSTE reflects RV function in chronic asymptomatic alcoholics more reliably as compared to conventional ultrasonic methods.

Alcoholic damage in ventricular pathology is generally considered to consist of both diastolic and systolic dysfunction. RV diastolic dysfunction is possibly related to myocardial relaxation and myocardial stiffness. Pathological changes are reflected mainly in myocardial conditions and myocardial fibrosis. Ethanol does affect tissue remodeling and causes ACM [35, 36], which is accompanied by cellular apoptosis and myocardial fibrosis [37]. In chronic asymptomatic alcoholics, levels of anti-apoptotic protein were lower, and the apoptotic index higher. Therefore, the active relaxation function was impaired. Myocardial interstitial fibrosis increases myocardial stiffness, which contributes to RV dysfunction in passive relaxation, and poor prognosis of patients with DCM. Collagen production is higher in ACM patients, which may contribute to progressive ventricular dilation and dysfunction in patients with dilated cardiomyopathy [38].

Alcohol injury may cause severe cardiac dysfunction in chronic asymptomatic alcoholics [39, 40]. The present contemporary view is that ACM is manifested structurally by increased volume and hypertrophy of the LV [41], and ethanol and its metabolite acetaldehyde are thought to be cytotoxic to myocytes. The deleterious effects caused by chronic alcohol intake include activation of apoptosis, dysfunction of intracellular organelles, myofilament alteration, and disorder of intracellular calcium homeostasis, which has a key role in alcohol-induced cardiomyopathy. The deleterious effect of excess alcohol is well established in the pathogenesis of myocardial fibrosis, disruptions in the myofibrillar structure, and loss of cardiac contractility, with concomitant reduction in pump function, decompensated by lowering of cardiac output [15, 42].

Ventricular interdependence has an important role in the pathophysiology of RV dysfunction [43]. Interdependence is mostly through the common wall, the interventricular septum. Failure of the left heart could affect function of the RV, and has even been reported to lead to RV failure [44]. In our study, LV longitudinal strain was less in both Group B and C than Group A and the control . Less strain reflects decreased cardiac function. LV dysfunction may influence RV function and further aggravate RV failure. While few studies have reported the influence of right heart function on LV, there is consensus that RV dysfunction is a predictor of poor outcome in the setting of LV failure [45].

Because most heavy drinkers remain asymptomatic in the early stages of disease progression, early detection of morphological changes may allow early intervention in the pathogenesis of cardiovascular dysfunction [46]. The results of our study with chronic asymptomatic alcoholics suggest that changes in RV pathology may cause attenuation of myocardial function and reduction of RV longitudinal strain. As seen in our observational study, the risk of dysfunction correlates positively with the amount of alcohol consumed. Among the groups of chronic asymptomatic alcoholics in the present study, lower systolic and diastolic function of all RV free wall segments relative to the control group was most significant in Group C.

Murata et al. [47] used 2DSTE to measure longitudinal systolic myocardial strain at the base of the RV free wall and suggested that the markedly reduced longitudinal strain in the systemic RV should be interpreted as a decrease in RV myocardial contractility. In our study, consistent with other findings [48], lower longitudinal P in the context of reduced S and SRs of the RV free wall suggests dysfunction in this patient population. TAPSE is another reliable index of RV systolic function [49]. The significant decrease of TAPSE in Group C also can be used for evaluation of RV systolic dysfunction. In addition, we found a significant negative correlation between global RV 2DSTE parameters S, SRs, and TAPSE consistent with previous reports [50, 51], which further illustrates that 2DSTE is effective as a method for detecting RV dysfunction. However, compared with 2DSTE, TAPSE is relatively load- and angle-dependent, and may be influenced by the LV. Moreover, TAPSE does not reflect segmental RV function and contractility [52]. 2DSTE can elucidate regional and global RV function, overcoming most of the limitations of TAPSE, because it is angle- and load-independent.

Notably, 2DSTE is strongly associated with RV contractile function confirmed by magnetic resonance imaging (MRI) [53–55]. As well, it has well-characterized specificity and sensitivity, and is a non-invasive imaging modality. Taken together, our results suggest that 2DSTE should be considered for detecting longitudinal myocardial systolic and diastolic RV function.

### Clinical implications

For the assessment of RV function, 2DSTE offers improvements over conventional ultrasonic evaluation. The results of this study suggest that 2DSTE can be applied in the evaluation of RV function in chronic asymptomatic alcoholics. However, further studies are warranted to fully elucidate the clinical benefit of this imaging modality.

### Limitations

The present study has several limitations. Firstly, it was conducted using a small number of male subjects, leading to uncertainty in extrapolation of these results across diverse sub-populations. Another limitation is that 2DSTE requires acceptable 2D image quality. Poor 2D image quality could confound interpretation of the results and negatively influence predictive validity of this imaging modality. In addition, several studies concluded that real-time 3-dimensional echocardiography had higher discriminating power than 2DSTE [56]. To test and verify the study, comparison with MRI and CT evaluation is necessarily. However, recent studies have shown a positive correlation between 2DSTE and MRI [53–55]. Therefore, larger studies are needed to study 2DSTE in clinical practice.

## Conclusions

Alcohol may increase the risk of RV diastolic and systolic dysfunction and ethanol-induced alterations in early cardiac function have the potential to develop into ACM. Although conventional echocardiography fails to detect RV function at an early disease stage, 2DSTE is a novel and sensitive technique to assess RV function. Our study demonstrates that 2DSTE is a promising imaging modality to detect early changes in cardiac function in chronic asymptomatic alcoholics. Future confirmatory studies are warranted to further evaluate RV function using 2DSTE in this patient population.

## Abbreviations

2DSTE: two-dimensional speckle-tracking echocardiography
ACM: alcoholic cardiomyopathy
DCM: dilated cardiomyopathy
EF: ejection fraction
LV: left ventricle
MRI: magnetic resonance imaging
RV: right ventricular
TAPSE: tricuspid annulus peak systolic excursion
TDI: tissue doppler imaging
